# Toe Necrosis: A Surprising Herald of Antineutrophil Cytoplasmic Antibody-Associated Vasculitis

**DOI:** 10.7759/cureus.96560

**Published:** 2025-11-11

**Authors:** Anil Regmi, Payal Shukla, Pragya Karki, Deya Obaidat

**Affiliations:** 1 Internal Medicine, Parkview Health, Fort Wayne, USA; 2 Internal Medicine, Nepalese Army Institute of Health Sciences, Kathmandu, NPL; 3 Rheumatology, Parkview Health, Fort Wayne, USA

**Keywords:** anca, myeloperoxidase antibody, rituximab, toe necrosis, vasculitis

## Abstract

Systemic vasculitides can affect any type of blood vessel and can present with various signs and symptoms depending on the site and type of affected blood vessels. The antineutrophil cytoplasmic antibody (ANCA)-associated vasculitis (AAV) can affect small- to medium-sized vessels. It can involve various organs and present with varying clinical and pathological features. Although rare, it can affect the blood vessels of the toes, leading to distal ischemia and gangrene. A 74-year-old woman with multiple comorbidities presented with symptoms of chronic nasal congestion, joint pain, and neuropathy, prompting autoimmune evaluation. Due to positive antinuclear antibody and perinuclear ANCA titer, initial treatment with low-dose prednisone and methotrexate was introduced. However, later during the course, she developed bilateral toe necrosis despite being on treatment. The workup for neurological and cardiovascular causes for the toe necrosis was negative. The patient ultimately required bilateral metatarsal amputation. Post-surgery, she was treated with rituximab and prednisone, which resulted in improvement in vasculitis. This case highlights the importance of recognizing rare presentations of AAV to enable timely intervention and prevent severe complications.

## Introduction

Vasculitis is defined as the inflammation of the walls of any type of blood vessel, resulting in the destruction of blood vessels and impairment of blood supply to the target organ [1,2]. The pathological hallmark of systemic vasculitides (SV) is infiltration of the vessel wall by inflammatory leukocytes, which can lead to ischemia and necrosis of the blood vessel wall [3]. Depending on the site and type of blood vessels affected, a wide range of clinical and pathological features are present [3]. According to the 2012 International Chapel Hill Consensus Conference Nomenclature of Vasculitides, the names of the vasculitides that were adopted include large-vessel vasculitis, medium-vessel vasculitis, small-vessel vasculitis, variable-vessel vasculitis, single-organ vasculitis, vasculitis associated with systemic disease, and vasculitis associated with probable etiology [4].

Antineutrophil cytoplasmic antibody (ANCA)-associated vasculitis (AAV) is a subgroup of SV. It is a group of small- to medium-vessel vasculitides involving various organs, most commonly the respiratory tract and kidneys [2,5]. There are three phenotypes of AAV, namely, granulomatosis with polyangiitis (GPA), microscopic polyangiitis (MPA), and eosinophilic GPA (EGPA), differentiated based on clinical and pathological features [6]. All three phenotypes can present with acute kidney injury and pauci-immune glomerulonephritis. GPA and EGPA more commonly affect the upper respiratory tracts, while MPA more commonly affects the kidneys and lower respiratory tracts [6]. According to some published literature, uncommon presentations of the AAV include pancreatitis, axillary lymphadenopathy, usual interstitial pneumonia, cystic lung disease, and splenic infarcts [7,8]. Rarely, AAV can manifest as distal ischemia and gangrene [9,10]. This case study discusses a rare case of AAV manifesting as toe necrosis, highlighting a rare clinical presentation of AAV and the complexities of its diagnosis and management.

## Case presentation

A 74-year-old woman with a past medical history of hyperlipidemia, hypothyroidism, diabetes mellitus, and hypertension presented to the rheumatology outpatient clinic with symptoms of chronic nasal congestion, periorbital headaches, shortness of breath, joint pain, and neuropathy, prompting an autoimmune workup. Her initial labs showed normal C-reactive protein, elevated erythrocyte sedimentation rate, elevated myeloperoxidase (MPO) antibody, positive antinuclear antibody, and perinuclear ANCA titer (Table 1). Other evaluations for lupus, hepatitis, and tuberculosis were negative (Table 1). Initially, the patient was started on a treatment with low-dose prednisone and methotrexate.

**Table 1:** Initial laboratory workup results. CRP = C-reactive protein; ESR = erythrocyte sedimentation rate; ANA = antinuclear antibody; ANCA = antineutrophil cytoplasmic antibody; p-ANCA = perinuclear antineutrophil cytoplasmic antibody

Tests	Result	Reference
CRP	1.1 mg/dL	0–0.9 mg/dL
ESR	37 mm/hour	0–20 mm/hour
ANA titer	1:80	<1:80
ANCA titer	p-ANCA positive titer >1:640	<1:20
Myeloperoxidase antibody	107	0–20.9
Lupus screening tests	Negative	-
Tuberculosis spot test	Negative	-
Acute hepatitis panel	Negative	-

**Figure 1:**
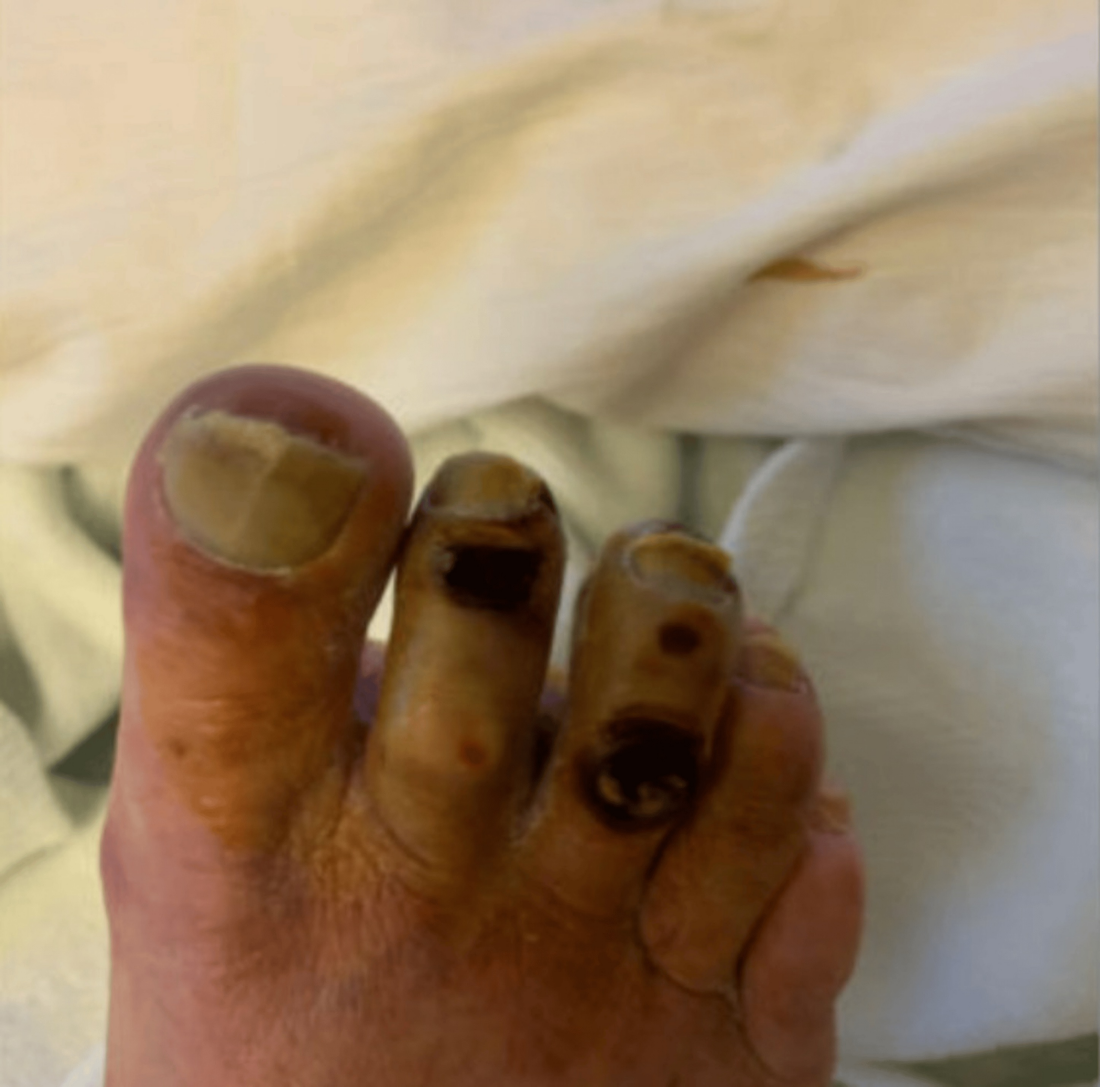
Early stage of necrosis of the second and third toes of the right foot.

**Figure 2:**
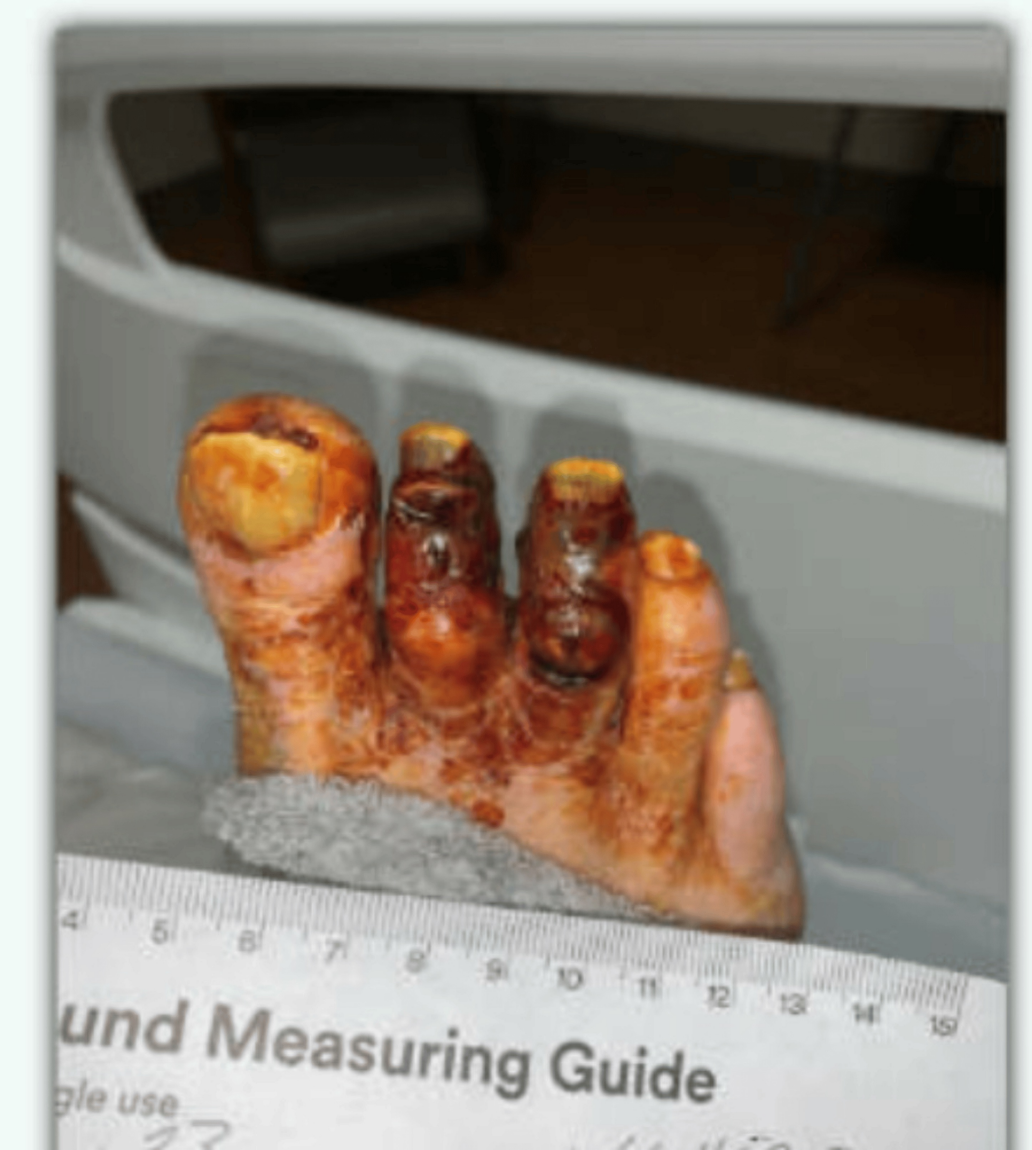
Progression of necrosis of the second and third toes of the right foot.

**Figure 3:**
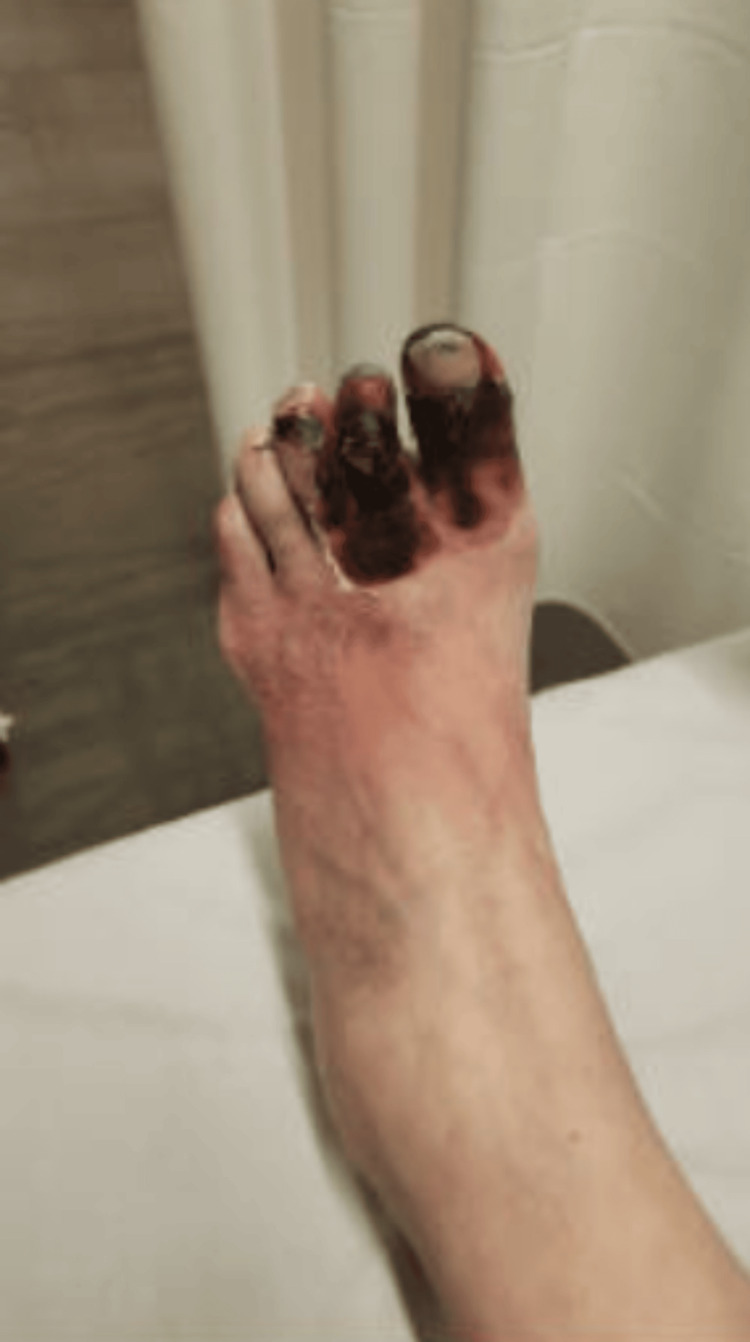
Necrosis of the first, second, and third toes of the left foot.

**Figure 4:**
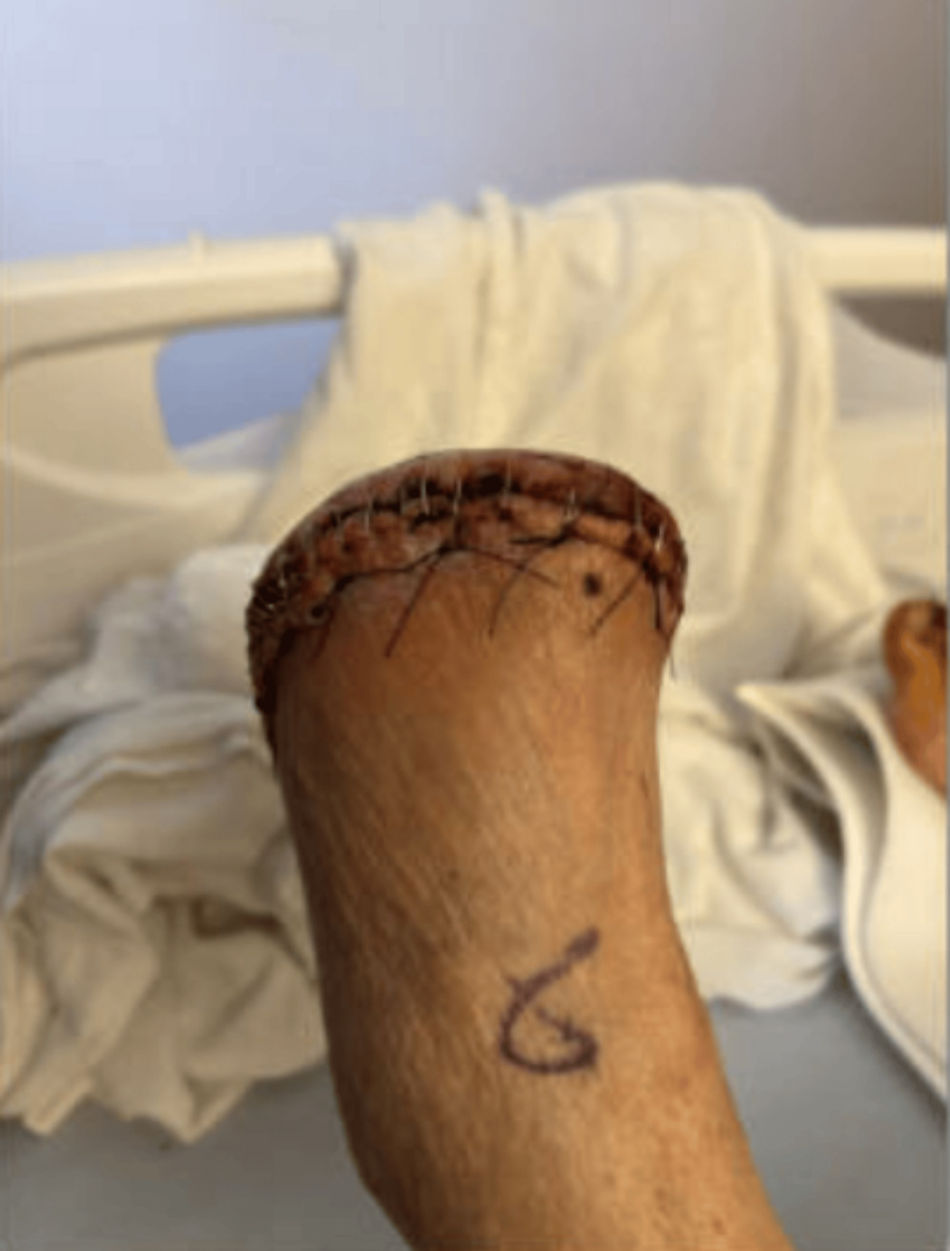
Status post-metatarsal amputation of the left foot.

A subsequent visit revealed new symptoms of foot drop, pain, and bluish discoloration over the toes bilaterally. Pulses over the bilateral dorsalis pedis arteries were intact. Symptoms were attributed to mononeuritis multiplex, and the patient was urgently referred to Neurology for electromyography. Additionally, a transthoracic echocardiogram, a CT angiogram of the lower extremities, and ankle-brachial index testing were performed to evaluate possible thromboembolic sources of her new symptoms. All of the workups were unremarkable. Treatment with high-dose prednisone and rituximab was started. Despite treatment, her clinical status worsened, and she developed toe necrosis bilaterally (Figures 1-3). Unfortunately, she required transmetatarsal amputation of both feet despite promptly starting her on an intravenous heparin drip (Figure 4). A biopsy obtained from the amputation sample revealed severe necrosis, acute-on-chronic inflammation, and granulation tissue. The patient was ultimately diagnosed with AAV, most likely GPA, due to positive MPO antibody. Following the amputation, she continued therapy with rituximab every six months along with a tapering dose of prednisone. Her symptoms gradually improved. Other than residual sinus-related symptoms and occasional joint discomfort, no active vasculitis was detected in subsequent visits. Laboratory markers of vasculitis improved. Although the patient still relied on a walker due to balance difficulties, she reported feeling stronger.

## Discussion

AAV is a group of diseases that involve severe, systemic, small- to medium-vessel vasculitides [11]. They are characterized by the generation of autoantibodies against the neutrophil proteins, leukocyte proteinase 3 (PR3-ANCA), or myeloperoxidase (MPO-ANCA) [11]. As discussed above, there are three main subgroups of AAV, namely, GPA, MPA, and EGPA, with prevalence varying in different parts of the world [6]. Clinically, AAV can affect multiple organ systems, such as the lungs, kidneys, skin, and nervous system [11]. However, the upper and lower respiratory tracts and kidneys are the most commonly and severely affected organs by these disorders [11]. Symptoms of upper respiratory tract involvement include nasal ulceration and nasal discharge, which can be bloody, sinusitis, or chronic otitis media [12]. Similarly, patients may develop lung nodules, alveolar hemorrhage, as well as pulmonary fibrosis in case of lower respiratory tract involvement, while renal involvement may present as hypertension, new-onset proteinuria, hematuria, and development of rapidly progressive renal failure [12]. The 2022 American College of Rheumatology/European Alliance of Associations for Rheumatology classification criteria for AAV exhibit robust performance metrics and have been validated for research use [13].

Some cases of small-vessel vasculitis may not have typical symptoms of upper and lower respiratory tract or renal involvement at the onset of the disease, thus delaying the diagnosis of severe disease. A similar example is presented in this case study, where the patient had bilateral digital ulcers with very subtle nasal congestion as the only symptom of AAV. A biospy sample obtained from the amputation showed signs of vasculitis in the digital arteries along with areas of necrosis. Although the prevalence is very low (<1%), there are some previous cases of ANCA-associated vasculitis reported to have digital ischemia and gangrene [9,14]. It is important to recognize that there can be some diagnostic challenges when patients have atypical presentations. Atypical presentations of AAV occur in certain severe instances, one of which includes inflammation from the disease, causing thrombotic and necrotic lesions [15]. Active vasculitis, causing destruction of the medium-sized vessel or in situ thrombosis, is the proposed pathophysiological process that leads to ischemia and gangrene of the digits [9,10]. Initial presentation can be simply pain and swelling, but can eventually form gangrene and necrosis if left untreated. Thus, it is to be noted that digital ischemia and gangrene may either be initial symptoms or may present later during the disease progression as a complication. Active vasculitis can be identified in angiography as well as histologically [9]. Sometimes, complications may occur despite undergoing active treatment [9]. In complicated cases, treatment with thrombolytic therapy can be helpful but may be unsuccessful, ultimately requiring surgical intervention or amputation [9]. Being aware of such atypical presentations of AAV can carry a favorable outcome by identifying the disease and initiating treatment at earlier stages of the disease.

## Conclusions

AAV can affect multiple organ systems. Although it most commonly affects the respiratory and renal systems, digital ischemia and gangrene can also occur. This implies that AAV has a variety of manifestations, and there can be some uncommon presentations. Clinicians may often miss the rare presentation of AAV, or these presentations can be misdiagnosed. Focusing on the treatment with no definitive diagnosis may lead to the development of disease complications. These complications can result in significant disabilities. We discussed a rare case of digital ischemia in a patient with AAV, which resulted in multiple toe necroses, ultimately requiring bilateral metatarsal amputation. This case encourages high diagnostic vigilance among clinicians when a patient with vasculitides presents with tissue necrosis that is resistant to treatment. It is also important to note that despite clinical alertness, definitive diagnosis of these conditions can be challenging, as deep tissue biopsies often yield nonspecific results. Complete and detailed history taking, thorough clinical examination, full comprehensive workup, and clinical awareness are required to recognize rare, uncommon presentations. Thus, this case report is an attempt to make healthcare providers aware of this rare manifestation, encouraging the prompt initiation of treatment as indicated.
